# The Necessity of Ambiguity in Self–Other Processing: A Psychosocial Perspective With Implications for Mental Health

**DOI:** 10.3389/fpsyg.2018.02114

**Published:** 2018-11-05

**Authors:** Christophe Emmanuel de Bézenac, Rachel Ann Swindells, Rhiannon Corcoran

**Affiliations:** ^1^Institute of Psychology Health and Society, University of Liverpool, Liverpool, United Kingdom; ^2^Research Institute for Health and Social Change, Manchester Metropolitan University, Manchester, United Kingdom

**Keywords:** ambiguity, sense of self, attunement, joint action, mental health

## Abstract

While distinguishing between the actions and physical boundaries of self and other (non-self) is usually straightforward there are contexts in which such differentiation is challenging. For example, self–other ambiguity may occur when actions of others are similar or complementary to those of the self. Even in the absence of such situational challenges, individuals experiencing hallucinations have difficulties with this distinction, often experiencing thoughts or actions of self as belonging to other agents. This paper explores the role of ambiguity in self–other differentiation, drawing from developmental, psychodynamic, and neurocognitive perspectives. A key proposal is that engagement in contexts that make distinctions between self and other challenging yet necessary allow reality-testing skills related to agency to develop. Attunement in typical caregiver–infant interactions is framed as a safe but inherently ambiguous environment that provides optimal condition for the infant to develop a coherent self–other sense. Vulnerability to psychosis may be related to limited access to such an environment in early development. However, the perceptual, cognitive, and social skills that contribution to attribution are likely to be malleable following infancy and improve though opportunities for boundary play in similarly ambiguous settings. Using music-making to illustrate, we postulate that engagement in intricate joint-actions that blurs agentic boundaries can contribute to the continued development of an adaptive sense of self and other essential to healthy social functioning. Increased insight into the self–other ambiguity may enhance our understanding of mechanisms underlying “self-disorders” such as schizophrenia and eventually extend the range of social and arts-based therapeutic possibilities.

“The brain abhors ambiguity, yet we are curiously attracted to it”

Ramachandran and Rogers-Ramachandran, 2008

## Introduction

Being alive is to possess a boundary or membrane that delimitates the inside from the outside, regulating what is kept in and let out, as well as what is kept out and let in. Awareness of the dynamic relationships that exist between oneself, one’s surroundings, and other agents is a primary, on-going task of the perceptual system (Gibson, 1979; Critchley et al., 2004; Gallagher, 2005; Gallese and Sinigaglia, 2011; Damasio, 2012). But how do we do this? When we do things, how do you know that it is “*us*” as opposed to another that is doing it? In most situations identifying one’s own self as separate from surroundings and other individuals and being able to attribute behaviors and events to their respective sources seems relatively straightforward and an essential part of everyday actions and interactions. However, there are times when this task is challenging, where information for distinguishing self from other (non-self) is either reduced or cannot be identified. While perceptual challenges of this sort have commonly been associated with phenomena such as hallucinations and delusions, self-other ambiguity can also exist as a characteristic of the external environment. Drawing from perceptual and developmental psychology, neuroscience, and psychodynamic theory, we explore the interaction between individual and environmental factors, proposing that ambiguity plays an important role in the development of an adaptive, flexible, and coherent sense of self essential to mental health and wellbeing through life.

## Defining the Self

The sense of self (and of “other selves”) is a perceptual, cognitive, and conceptual organizing system by which we encounter the world (e.g., James, 1891; Rogers, 1961; Stern, 1985; Damasio, 2003; Baumeister and Bushman, 2011), which is central to any understanding of human psychology and mental health. Yet still, its conceptualization remains mired in a theoretical quagmire (Baumeister, 1987; Berrios and Markova, 2003; Guignon, 2004; Klein, 2012; Gallagher, 2013) because sense of self is a complex and multifaceted construct involving over-arching and over-lapping processes like consciousness, agency, memory, and social and cultural identity (Gallagher, 2000; Klein and Gangi, 2010; Leary and Tangney, 2011). Typically, however, theoretical accounts share a similar focus on the sensory-motor and mental processes which endow one with feelings of singularity, stability, and coherence as an individual human being (Siegel, 2001; Damasio, 2003). A distinction is also commonly made between a “minimal” or “core” self, accessible to immediate self-consciousness as moment-to-moment streams of multisensory, perceptual, and affective experience, and a narrative or “extended” self drawing, for example, on higher order mental representations and episodic memory (Stern, 1985; Siegel, 2001; Sass and Parnas, 2003; Gallagher, 2005; Klein and Gangi, 2010). The former – the main focus of the current paper – is an essentially embodied phenomenon encompassing a sense of body (bodily unity/coherence), ownership, and agency, which, crucially, allows the differentiation of self from other/environment. The latter encompasses a sense of self-identity and personhood often over a longer time-frame (Gallagher, 2013). Although discrete phenomena, theorists have emphasized the inter-dependency between these lower and higher order configurations, with multiple layers of self regarded as operating in parallel throughout life. For Stern (1985), for example, the achievement of a “core self” between 2 and 7 months of age not only sets the ground work for the subsequent emergence of “verbal” and “narrative” selves, but also establishes a sense of one’s self as a unified and integrated but separate being. Thus, this functioning core self is essential for maintaining mental health across the lifespan, keeping at bay feelings such as dissociation and fragmentation (Fink, 1988).

Aside from the identification of different types of self (Klein, 2012), researchers across domains have emphasized the inherent malleability of these differing “selves.” Anthropologists argue that definitions of self are, at least in part, culturally determined and vary across time and place, where sense of self is regarded as more or less fluid in distinct socio-cultural contexts (Baumeister, 1987; Guignon, 2004; Christopher and Hickinbottom, 2008; Benning, 2013). Childhood researchers likewise highlight how the emergence of a sense of self across developmental milestones and in tandem with wider developmental achievements occurs in transaction with necessary environmental inputs. While not denying the influence of genetic inheritance, the neurological/biological basis of one’s sense of self is shaped to a large extent through interactions in the infant’s social environment and interpersonal relationships (Damasio, 1999; Siegel, 2001; Schore, 2003, 2015; Tronick, 2007).

## Physical and Agentic Boundaries of Self and Other

From the very start of life, we learn that there are fundamental differences between the outcomes of our own actions and the outcomes that results from the behavior of others around us (see White, 1995, 50–56); we come to experience direct control over our actions and an ability to move and manipulate objects, surfaces, and even, eventually and to a certain extent, the actions and thoughts of other agents (Gibson and Pick, 2000, 160). This experience relies on knowing the boundaries of entities, where one thing ends and another begins. But how are such boundaries determined, particularly those that exist between self and other agents? Put in more concrete terms, how can sensory signals deriving from the presence or movements of one entity be disentangled from those belonging to another?

It has been argued that we do this by relying on invariants – gestalt-like regularities about the characteristics of animate and inanimate objects (Gibson, 1966; Stern, 1985; Bregman, 1994; Palmer, 2003). For example, an object tends to possess unity: its parts move together when they move or are moved. The stimuli generated by such movement, whether picked up in the form of sound, light, taste, or pressure, are therefore likely to seem coherent and perceived as belonging together (Rock and Palmer, 1990). The sounds of a person speaking, for instance, derive from a similar location and change together gradually rather than suddenly, sharing a common temporal and intensity structure. Sounds that do not share this coherence, such as an utterance that is suddenly much louder or derives from a different location, suggest the presence of another speaker. Therefore, while coherence is the norm (an invariant) within entities, incoherence is expected between them and is therefore used to mark their distinction. Separate entities segregate by virtue of their distinctness: they occupy different locations to other nearby objects and surfaces and tend to move independently, generating stimuli with temporal and intensity profiles that are incoherent/mismatched in relation to one another.

**FIGURE 1:**
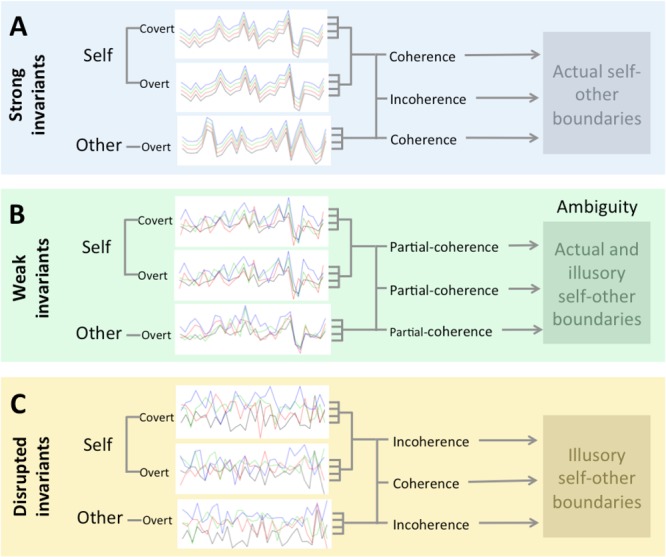
Defining ambiguity between self and other boundaries. Time-series represent sensory signals/stimuli (lines). These signals can either be overt (externally accessible, e.g., sound, vision, and smell) or covert (inner-body signals accessible to self, e.g., proprioception, volition, action prediction, and emotion sensations). All signals can vary in multiple ways, e.g., modulating in intensity, timing, shape, location, and form over time. Coherence between such signals within entities (e.g., self) and incoherence between entities (self and other) is the default (invariant) and allows us to easily perceive the world as it is, i.e., the actual physical and agentic boundaries that exist between entities **(A)**. Invariant can be disrupted: incoherence within and coherence between entities, resulting in illusory boundaries **(C)**. Ambiguity **(B)** emerges when invariants are weakened to the extent that signals are partially coherent/incoherent within and/or between entities. Both actual and illusory boundaries (physical and agentic) are available for perception and the perceiver can switch between these alternatives.

As the above examples indicate, the boundaries of entities and, in particular, those of animate entities are determined by how stimulus features such as intensity, timing, and shape vary over time (Stern, 2010). Stimuli with matching profiles are perceived as belonging together and having derived from a coherent entity (Figure 1). As Stern (1985, 154) argues, such dynamic features are amodal in the sense that they describe variation in signals picked up in all sensory modalities, including those originating from within the body (also see Lewkowicz and Turkewitz, 1980; Kuhl and Meltzoff, 1982; Stein and Meredith, 1993; Pascual-Leone and Hamilton, 2001). For example, a sudden increase of intensity does not only refer to changes in aspects of sound, light, touch, and smell, but also to variation in more covert signals such as proprioceptive feedback or an affective sensation such as fear. Significant to agency, it can also describe the content of sensorimotor as well as longer-term predictions that one forms about the results of actions as they unfold over time.

Coherence between stimuli originating outside and inside the body provides essential information about which entity corresponds to self as opposed to other, contributing to the feeling of self-agency, the sense that “I am in control and the source of my thoughts and actions” (Gallagher, 2000). Control is therefore not only central to the experience of being an agent, but also key to understanding the dynamics of social interaction. Changes in the sense of agency have been described as an alteration of the perceived control over the relationship between actions and outcomes (Moore et al., 2009; Desantis et al., 2011). It derives in part from basic physiological systems of the body in relation to sensory stimuli (Von Holst, 1954; Jeannerod, 2003; Poulet and Hedwig, 2007). As the “comparator model” suggests, events that match the predicted consequences of action are experienced as belonging to self (prediction error is small), while mismatch (or large prediction error) is attributed to an external cause (Gandevia and Burke, 1992; Wolpert and Miall, 1996; Frith et al., 2000; Trinity and Sommer, 2008). In other words, an invariant of agency is that the difference between predicted variation in the intensity, timing, and shape of an action and the actual intensity, timing, and shape of the resulting stimuli is likely to be small when that action belongs to self, compared to when it belongs to another individual. Stimuli belonging to self as opposed to another agent are likely to vary with a number of other covert (internally derived) signals not directly accessible to other individuals. The latter could include signals that precede, accompany, or follow actions, such as volition (as well as higher order intentions and goals), action prediction, sensory and proprioceptive feedback, affective sensation, as well as the evaluation of past behavior (Stern, 1985; Gallagher, 2000; Wegner, 2002; Wegner et al., 2004).

Although this paper is primarily concerned with lower level perceptual processes, it is worth noting that the sense of agency implicates control specified at different hierarchical levels. Synofzik et al. (2008) make a distinction between a low-level, pre-reflective “feeling of agency” and a more explicit “judgment of agency” where self–other attribution exists at a higher conceptual level. While neurocognitive agency research has tended to either focus on internal sensorimotor processes (Blakemore et al., 2002; Haggard, 2005) or external situational factors (Wegner, 2002, Wegner, 2003), social psychology literature has addressed higher level attribution (Kassin et al., 2010). Experimental measures used to investigate dimensions of agency are therefore diverse, ranging from explicit psychometric and verbal reporting to implicit paradigms such as intentional binding (Haggard et al., 2002). Together these findings suggest that the sense of agency is influenced by varied cues and by an ability to integrate them with one another (Moore and Fletcher, 2012; Kranick and Hallett, 2013).

## Ambiguity Between Self–Other Boundaries

In most situations, stimulus features either vary coherently or incoherently in relation to one or more entities, with available sensory modalities pointing in the same direction. This enables boundaries that exist between entities including self and other and the control that these have over occurrences to be clearly defined. However, rather than seen as binary opposites, there is evidence that coherence and incoherence are better conceptualized as opposite ends of a continuum (Farrer et al., 2003). With effort, an agent can generate stimuli that are partially incoherent, adopting a characteristic more commonly associated with a number of unrelated objects or agents. A ventriloquist, for example, rapidly changes the quality of her/his voice to promote the illusion of being in the presence of more than one individual (Howard and Templeton, 1966; Soto-Faraco et al., 2002; Alais and Burr, 2004). Autonomous agents can also act with partial coherence in relation to one another, generating stimuli that vary with a degree of synchrony more typical of a single agent, such as a group of individuals marching together (McNeill, 1997).

Furthermore, actions belonging to self can display a degree of incoherence between internal signals such as action predictions and external feedback: consider the experience of using a malfunctioning computer mouse where visual feedback does not correspond to intended and performed actions. Experiments that set up to investigate mechanisms of agency typically introduce spatial or temporal distortions to the outcome of a participant’s actions to manipulate the authorship that a person feels over that action (Blakemore et al., 2000; Franck et al., 2001; Sato and Yasuda, 2005; Farrer and Franck, 2007). As action–outcome discordance increases (e.g., using delay or spatial displacement), the participant is more likely to disown the sensory feedback, attributing it instead to an external cause. The opposite is also possible. Signals related to actions of self, including intentions, predictions, proprioception, and affective sensation, can be coherent with externally generated stimuli. This is demonstrated by Wegner et al. (2004) who found that having thoughts that happened to be coherent with an action performed by the experimenter made participants more likely to experience ownership over that action – a phenomena that they called vicarious agency.

Invariants can, therefore, be weakened. In summary, although it is the case that changes in intensity, timing and shape are expected to be coherent within but not between entities, and interoceptive–exteroceptive coherence is the norm for self-generated but not for externally generated actions, such regularities can be partially violated. This can result in the emergence of conflicting groupings, with some features pointing toward illusory boundaries or causal relationships and others specifying “reality” – the actual boundaries or causal relationships that exist in the world. Informational conflicts may cause us to momentarily misperceive stimuli deriving from multiple agents as belonging to a single coherent agent (e.g., the marching group) or, vice versa, stimuli from a single agent as belonging to multiple agents (e.g., the ventriloquist). In relation to agency, we may also feel that our own thoughts, emotions, and actions and their consequences belong to another individuals. The Ouija board game exemplifies the latter: the combined force that a number of participants exert on the centerpiece makes individual contributions difficult to ascertain, promoting the misattribution of movement to an external force (under-attribution to self) (Ansfield and Wegner, 1996). Conversely, the illusory experience of controlling externally caused events can also occur (over-attribution to self). One example is a conductor who experiences a high degree of coherence between his/her actions (and associated internal signals such as volition and sensorimotor predictions) and the sensory outcomes of orchestra members’ collective actions (Epstein, 1987). However, given that invariants can never be completely violated, such misperceptions are fleeting and rarely complete. Even in controlled settings where there is an explicit attempt to promote misperceptions, there is generally always sensory information available that continues to point toward the state of the world as it is (Gibson, 1966, 1979).

Ambiguity emerges when invariants are weakened to an extent that multiple conflicting groupings are perceivable, whether these are actual or illusory entity boundaries or causal relationships (see Ernst and Bülthoff, 2004; Roach et al., 2006). In such contexts, there is insufficient information available to determine which alternative is preferred due to a balance between coherence and incoherence specified by dynamic features of stimuli across sensory modalities (see Rimmon-Kenan, 1977, 17). The perceiver is driven to explain or find meaning in the experience. They conduct a series of overt and covert reality tests when faced with inadequate or conflicting information, such as head movements or attention shifts, to search for “information that will reinforce one or the other alternative” (Gibson, 1966, 303–304). As Ramachadran and Rogers-Ramachandran (2008) contend, “the brain abhors ambiguity, yet we are curiously attracted to it” – an attraction which may have evolved due to the need for organisms to have an incentive to uncover objects, such as predators, from complex environments (Ramachandran and Hirstein, 1999). Where disambiguating information is limited, the perceptual process is necessarily prolonged, as exemplified by the incessant alternation between the possible alternatives that characterize the viewing of bistable images (e.g., duck or rabbit) (Wernery, 2013). Ambiguity in effect disrupts habitual perception and subjective experience – one is compelled to look and listen again (Rose, 2004, 148–149). Artists, writers, and performers have long understood and exploited this (Rimmon-Kenan, 1977, 229). Gibson (1979, 44) explains that picture-makers enhance esthetic experience by introducing “a discrepancy of information, an equivocation, or contradiction in the same display,” while restricting the amount of additional information that can be gathered through objective scrutiny.

## Ambiguity and the Developing Self

Ambiguity in the perception of entity boundaries, particularly relating to self and other, is a pervasive feature in accounts of infantile experience and early processes of separation, individuation, and sensory integration. Although rudimentary processes of self–other differentiation including self-agency are commonly thought to be evident from birth (Meltzoff and Moore, 1995; Rochat, 2003), it is generally accepted that an infant’s sense of self emerges at psychological and neurobiological levels through its relationships with others (Bowlby, 1973; Damasio, 1999; Siegel, 2001; Perry, 2002; Tronick, 2007; Schore, 2015). This is supported by evidence for shared neural networks for processing self and other in multiple brain areas including cortical midline, frontal, and parietal structures (reviewed in Frith, 2007; Lieberman, 2007; Uddin et al., 2007).

Following the physical separation that begins from the first moments of post-natal life, the human baby is primed to respond to and attract the social contact required for its survival and development (Stern, 1985; Siegel, 2001; Schore, 2003, 2015; Tronick, 2007). The caregiver’s first role is not only to provide physiological protection and nourishment but also emotional containment and feelings of self-coherence by regulating his or her changing levels of arousal associated with internally and externally derived sensations. Responding to fluctuating sensorimotor and affective cues, the caregiver seeks, for example, to calm a distressed or over-excitable infant, entertain a passive infant, and temporarily withdraw when an infant is overstimulated, while also attributing intent and agency to such covert behaviors (Brazelton et al., 1974; Gergely et al., 2002; Fonagy, 2003). Ensuring the infant enjoys sufficient periods of calm to engage in self-perception-based exploratory play also contributes to the early sense of self by means of “the intermodal calibration of the body” (Rochat, 1998).

Drawing on micro-observational studies examining infant–caregiver interactions on a moment-to-moment basis, Stern’s (1985) work emphasizes the importance of attunement processes, achieved as the caregiver matches to or mirrors the dynamic features of behaviors, particularly those expressing variation in an infant’s internal affective states. By minimizing the separateness that typically exists between entities, caregivers intuitively seeking to reduce a younger baby’s potential frustrations and distress allow the infant to experience an illusion of oneness and pleasurable feelings of agency and extended control over surroundings (Winnicott, 1960, 1971; Gergely and Watson, 1996; Fosha, 2001; Glover, 2009; Dowds, 2014). An example of this is a parent who attunes to the intensity, timing, and shape of a baby’s animated movements (e.g., raised and lowered arms) by means of accompanying vocalizations (e.g., “wheee!”) matching the rise, fall, and overall excitation levels inherent in the infant’s gestures.

As this scenario exemplifies, empirical work suggests that rather than solely imitating the infant’s behavior, the caregiver, over time, begins to translate the contours of that behavior into an alternative sensory modality (Jonsson et al., 2001; Crown et al., 2002; Beebe et al., 2010). In perceptual terms, this transformation not only emphasizes the dynamic features of behavior (given that these remain the same), but also provides the infant with opportunities to learn to, weigh up, and synthesize information from differing modalities that specify internal and external states. That dynamic features are matched in one modality but not another functions to educate attention and help promote sensory integration – the development of a normal sense of self depends on emerging abilities to integrate multisensory input (Postmes et al., 2014). According to Stern (1985), however, it does much more than this in that it also helps the infant to grasp that the caregiver is not only able to mimic his or her literal behavior, but has understood the affective sensations underlying it. Ultimately, this conveys to the infant that external actions but also internal subjective states of mind in the self and other can be known and shared – an important step in the acquisition of what has variously been called a mentalizing capacity (Fonagy et al., 2004; Frith and Frith, 2006), theory of mind and empathy (Premack and Woodruff, 1978; Baron-Cohen, 1991; Corcoran et al., 1995).

Given that some aspects of behavior are attuned to, while others are not, and that dynamic features may be matched in one modality but not another, there will always be sensory information continuing to point to the caregiver–infant as separate, distinct entities. With reference to the illustration of cross-modal attunement above, while coherence or “oneness” is specified in the correspondence between the infant’s motions and caregiver’s sounds, there is a mismatch between the agents’ arm movements which make conflicting cues available to the infant. The fact is there is no such thing as a perfect attunement with a degree of incoherence always pointing toward to divergences between infant and caregiver as distinct agents. Indeed, moments of misattunement, whether purposeful or unintentional (the over or under shooting of behavior contours), are as necessary as episodes of attunement to help the growing infant identify and integrate the invariants that distinguish its experience of itself from an other as an embodied, feeling, and, eventually, thinking being (Stern, 1985; Fonagy and Target, 1996; Tronick, 2007). As clinicians and researchers have noted, too much, as well as too little attunement coherence are both detrimental to developmental outcomes in this regard (Stern, 1985; Jaffe et al., 2001; Fonagy, 2003). There may be a critical tipping point that is key to life chances and outcomes between enough, not enough, and too much attunement.

The overall implicit goal is healthy ambiguity: a balance emerges over shorter and/or longer periods of time in the fluctuation of attunement/misattunement, between stimuli pointing toward caregiver–infant togetherness (coherence) and separateness (incoherence). In the “wider-world” situations that do not allow self and its outcomes to be distinguished from surroundings can be dangerous and perceived as such. The infant’s gradual awareness of his/her separateness and agentic limits (the reality of self/other bounds) is likely to rouse unpleasant feelings associated with helplessness and dependency. The sensitive caregiver responds by creating a safe yet sufficiently motivating context in which the infant is invited to explore and *play* with the boundaries between self and other and, at the same time, his or her own internal and external experiences. While the precise manner in which the caregiver facilitates such implicit learning varies across development, perceptual play in a variety of forms continues to promote awareness of self–other boundaries by bridging the subjective world of the imagination and the outer world of people and things (Winnicott, 1971; Milner, 2010). Being continuously called upon to compare fluctuations in the degree of coherence that exists between entity boundaries and internally and externally derived signals provides the individual with optimal conditions for honing the perceptual, social, and cognitive competencies required for a functioning sense of self and of agency. As Winnicott, Milner, Segal, and others contend (see Glover, 2009), this in-between space is also the basis of creativity and a capacity to symbolize (to be non-literal or pretend) in the context of verbal thinking and communication with others. In contrast to persisting romantic notions of creativity as the cathartic endeavor of a lone genius, these authors argue that it emerges not by losing touch with reality or with others, retreating into one’s inner world, but instead by an increasingly refined awareness of, and playful engagement with the boundaries between internal and external experience – through a fluid interplay between the two.

In brief, ambiguity provides a way of thinking about mechanisms relevant to early psychological development. Attunement–misattunement fluctuations in typical caregiver–infant interactions are framed as a safe but inherently ambiguous environment. The coherent sense of self and other develops through ambiguity by making self–other differentiation simultaneously necessary, motivating (often playful) and challenging.

## Implications for Mental Health

Closely allied to this facility to differentiate self and other is an ability to distinguish between what is real from what is imagined, made up, or simulated (Fonagy, 2003; Sutton-Smith, 2009). Ecological psychologists contend that the perception of the tangible, external world is always distinguishable from that of mental life such as dreams and hallucination, in that the latter does not yield additional information when subject to scrutiny or “reality tested” (e.g., scanning with eye, head, hand, and body movements). Gibson (1970) argues that the reason individuals experience hallucinations or “psychedelic experiences” as external reality reflects either an inability or disinclination to apply the necessary perceptual tests, for example, when under the influence of drugs or during periods of psychological distress. This is supported by theoretical models that understand psychosis as a deficit in information processing (Bellack et al., 1990; Green and Horan, 2010; Savla et al., 2012; Aleman, 2014) or impaired salience assignment also associated with hyperdopaminergic neural state (Kapur, 2003; Winton-Brown et al., 2014). Although conflicting cues in the environment are what motivates the search for additional information, ambiguity can stimulate anxiety, impacting on the efficiency and conclusions of ongoing thinking as well as the capacity to apply appropriate reality tests when required (Eysenck and Calvo, 1992; Beck and Clark, 1997; Maule et al., 2000). One result can be a tendency to over-rely on biases or prior knowledge that no longer apply (Corcoran et al., 2006; Bennett and Corcoran, 2010), with an urge to adopt new certainties too quickly, before a new pattern has had the chance to emerge (Bion, 1970, 124). Notably, an inability to tolerate ambiguity and a proclivity to jump to conclusions in uncertain contexts has been consistently linked to psychopathological disorders including psychosis (Budner, 1962; Linney et al., 1998; Colbert and Peters, 2002; Grube, 2002; Van Dael et al., 2006; Broome et al., 2007; Garety et al., 2011).

Social psychologists have examined how individuals respond to socially ambiguous situations (Heider, 2013). In this literature, the term “attribution” is used to explain how individuals find causes for their own or others’ behavior (Kelley, 1967). Particularly when outcomes of behavior are predictable, causes can be ascribed to internal or dispositional factors such as an individual’s (or a group’s) motives, beliefs, and personality traits. They can also be ascribed to external, situational forces that go beyond one’s control (Moskowitz, 2005; Kassin et al., 2010; Heider, 2013). Attribution theory assumes that people make causal inferences rationally by assessing the wider social context of behavior. However, attributions do not always accurately reflect reality. In socially ambiguous contexts, they are particularly susceptible to systematic biases. Erroneous assumptions about a person’s behavior, for example, can be based on whether or not outcomes are desirable and on social groupings (in-group/out-group) (Forsyth and Donelson, 1987). There is a tendency to see success as related one’s own disposition with failure ascribed to situational factors (blaming surrounding), while the opposite is the case when assessing others, particularly those outside the group (a self/group-serving bias) (Myers and Smith, 2012). Although ambiguity calls for a search for further information, individuals also tend to also gloss over informational gaps when attributing behaviors to others, giving undue salience to readily available information as an alternative to continued reality testing. Many dimensions are thought to influence attributional style including culture (e.g., individualistic vs. collectivist societies), language, beliefs, as well as clinical conditions such as paranoia and depression. Individuals vulnerable to depression, for example, tend to attribute their own failures to internal, stable, and global factors (Abramson et al., 1978; Pyszczynski and Greenberg, 1987). A hostility perception bias – the tendency to experience others’ intention as hostile in ambiguous settings – has been associated with paranoia, traits of emotional dysregulation, and theory of mind difficulties in both clinical and non-clinical populations (Combs et al., 2009; An et al., 2010; Jeon et al., 2013). Such biases may be seen as constituting a repertoire of adaptive and maladaptive strategies that people rely upon in social exchanges that almost always involve a degree of ambiguity. But are there ways of protecting against such biases or short cuts to continued thinking?

Early experience in a safe environment that allows actual and illusory boundaries to be evaluated and compared one with the other without confusing the two develops the ability and propensity to conduct appropriate reality tests on the corporeal and agentic boundaries of self and other. The ambiguous play facilitated by the caregiver enables the infant to experiences different states of self and other over time, leading to a sense of self that is more resilient and adaptive to the internal and external changes that occur throughout life. There is less need to resort to ready-made attributional biases. By contrast, situations where reality is always or never clear-cut, where invariants specifying entity or agency boundaries remain unchallenged and rarely come into conflict, are not likely to afford such opportunities for psycho-social development. Fonagy (2003) contends that the most crucial outcome of a secure attachment relationship in childhood is the ability to distinguish between and realistically appraise self and other, rather than the usually cited engendered feelings of safety and self-confidence *per se*. Individuals who do not acquire the competencies required to maintain a distinction between representations of self and other – where the actions and/or feelings of one are habitually confused and misattributed to the other – may have to develop less adaptive strategies to amplify the differences between the two, through for example forms of social withdrawal or preoccupation with other. More generally, all mental illness may be viewed as the mind misinterpreting its own experience of itself and of other (Fonagy and Campbell, 2015), with a failure to establish developmentally appropriate constructs of self in the early years implicated in the etiology of various disorders (Fink, 1988; Kyrios et al., 2015). This is particularly apparent in agency-related phenomena such as hallucinations and delusions of control thought to be rooted in difficulties in differentiating between thoughts, intentions, and actions belonging to self from those belonging to others (Spence et al., 1997; Parnas and Handest, 2003; Sass and Parnas, 2003; Woodruff, 2004; Lindner et al., 2005; Ditman and Kuperberg, 2005; Frith, 2005; Bentall et al., 2007; Jeannerod, 2009).

Behavioral and neuroimaging research has begun to shed additional light on mechanisms underlying the sense of self and of agency (David et al., 2008; Nahab et al., 2011; Sperduti et al., 2011) and on the impact of development on such processes (e.g., Kircher and David, 2003). Early attachment experiences can positively or negatively shape genetically primed neural structures that underpin perceptual and cognitive organization of self (Bowlby, 1973, 1982; Siegel, 2001; Schore, 2003, 2015; Tronick, 2007). For example, a body of research has focused on experience-dependent maturation and stabilization of interconnections between the orbitofrontal cortex with cortical and sub-cortical areas in early life, and the essential role these associated pathways play in self-regulatory behavior and self-monitoring (reviewed in Schore, 2015). Likewise, a number of studies have shown that disrupting parent–infant interactions during early development can have significant impact on the development of the prefrontal cortex in humans and other mammals (reviewed in Kolb et al., 2012). This region, which has been associated with guiding motor, affective, cognitive, and social behavior over time (Wood and Grafman, 2003; Mitchell et al., 2005), is thought to have a prolonged, experience-dependent development, making it particularly susceptible to abnormal functioning as expressed in multiple neuropsychiatric disorders (Stuss et al., 2001; Tekin and Cummings, 2002; Braun and Bock, 2011).

While the development of a core sense of self/other in infancy is a specific keystone achievement associated with critical periods in infancy (Stern, 1985; Fink, 1988; Kyrios et al., 2015), a “mature” sense of self must be honed through life’s experiences. Indeed, the development of psychotherapeutic interventions attests to an understanding that enhancing self-awareness and adjusting to ever-changing realities is a long-term endeavor. As evidenced by research into behavioral and brain plasticity (Ponti et al., 2008; Pascual-Leone et al., 2011; Keller and Just, 2016), the manner in which a person perceives surroundings is always susceptible to learning and development, with encounters in the world presenting limitless opportunities for fine-tuning attention and sensitivity to novel or previously undetected information (Gibson, 1979, 254). Below we argue that contexts that blur the distinctions between self and other, inner and outer, reality and non-reality, such as artistic or esthetic pursuits involving intricate joint behavior, may be especially helpful in this regard. We suggest that in terms of cognitive and affective outcomes they are analogous to the caregiver–infant interaction processes by providing a route to guide reality monitoring reflexes and self–other attribution, leading to an adaptable sense of self. If correct, it follows that individual differences in self–other attribution and reality monitoring will be related to experience in these pursuits. Furthermore, long-term engagement in these pursuits may itself be predicted by the quality of early caregiver interactions.

## Ambiguity-Promoting Behaviors: the Case of Music-Making

In any social contexts, events tend to be co-produced, neither fully belonging to self or to other but resulting from the mutually regulated actions of both (Sebanz et al., 2006; Konvalinka et al., 2010; Badino et al., 2014). Interaction always involves, indeed demands, a weakening of invariants. The coherence that typifies stimuli deriving from an individual agent and the incoherence that comes to be expected between autonomous agents is partially reduced as participants match to the varying intensities, timings, shapes, or forms of each others’ behavior outcomes. In effect, this allows a quality of “we-ness” to emerge. Those involved momentarily function as a larger “whole” or system with its own emergent properties.

Music-making provides a particularly clear, though by no means unique, illustration of this. A musician, through thousands of hours of practice, learns to accurately coordinate highly intricate movements with those of others to produce specific auditory effects (Ericsson and Lehmann, 1996; Sloboda, 2000). The manner in which musical instruments are constructed and played indicates that controlling the degree of coherence and incoherence in sound (termed integration and segregation in this context) is of fundamental importance to all forms of music-making (Bregman, 1994, 458, 674). In contrast to most listening experiences where the goal is to detect actual sound-sources in the environment, music often tries to create illusory sources or what Bregman (1994, 460) calls “auditory chimeras”: “It [music] may want the listener to accept the simultaneous roll of the drum, clash of the cymbal, and brief pulse of noise from the woodwinds as a single coherent event with its own striking emergent properties.” This is achieved by going against invariants of coherence within and incoherence between entities or sound-sources. It is this skill that much of the effort of acquiring musical expertise is focused on – whether it be by learning to play in tune and in time with others or via the technical dexterity that enables an individual’s sounds to segregate while blending with sounds produced by other musicians.

The literal meaning of symphony is “sounding together” and there are many examples where the contributions of individual musicians are blurred by introducing coherence between separate entities. This is particularly evident in contexts such as choirs or percussion ensembles where sound-sources are similar to one another. In the gamelan traditions of Southeast Asia, for instance, two or more musicians commonly perform interlocking patterns designed to be heard as deriving from a single coherent sound source (Tenzer, 1998; Bakan, 2007). This illusion of oneness is made possible by virtue of the coherence that exists between the sounds generated by the two musicians: their close proximity to one another, the similarity of the timbres produced by their respective instruments, and in the coherence of intensity, timing, and pitch material between the contributions. Features specifying coherence, however, are at the same time balanced by cues that continue to point toward the incoherence that persists between autonomous agents both within and between sensory modalities. In other words it is still possible to see and partially hear that the two musicians are separate entities through (albeit slight) differences in spatial location, and in the variation of intensity, shape, and form associated with each agent. The fact that this is an effortful task for many of us might attest to the social processing default of the human brain.

Conversely, by introducing incoherence characteristic of stimuli belonging to more than one entity, whether it be through abrupt changes of pitch range, intensity, timbre, or spatial location, the sounds of a single musician (sound-source) can also split and be misperceived as deriving from separate entities. Partial segregation may be heard in many music traditions: examples include the abrupt changes of timbre produced by Chinese dizi flute music (Tsai, 2004), Mongolian diaphonic chatting (Lindestad et al., 2001), and the pseudo-polyphony in late Baroque music generated by rapidly switching between pitch range/register (Davis, 2006). Regarding the latter, Bregman (1994, 464) writes: “these alternations were not fast enough to cause compulsory segregation, so the experience was ambiguous between one and two streams.” Frequently in music partial incoherence within entities is combined with partial coherence between them, with sounds belonging to one musician made to segregate from other sounds generated by the same musician while merging with components of sound belonging to other musicians.

Music, among other intricate joint behaviors such as dance, theater, and certain sports (McNeill, 1997; Sebanz et al., 2006; Hove, 2008; Overy and Molnar-Szakacs, 2009; Pacherie, 2011), may therefore be seen as implicitly promoting ambiguous perception, ensuring that entity boundaries are sufficiently blurred and that an equilibrium is reached between multimodal sensory conflicts that point toward togetherness, on the one hand, and separateness, on the other. As also described in relation to attunement processes during early development (often described using musical metaphors, e.g., proto-musicality, Malloch and Trevarthen, 2009), going against invariants in this way promotes a drive to test reality characterized by shifts between actual and illusory boundary alternatives.

When a person is directly *involved in* ambiguity-promoting behavior, blurred boundaries extend not only to those between entities but also to feelings of agency. Take for example the experience of not being able to distinguish one’s own voice from those belonging to other group members when singing in a choir. One moment it might feel that another’s voice belongs to self (a case of over-attribution to self) and the next that one’s own voice is not of our own making (over-attribution to other). This involves semi-coherence between internally and externally derived signals: the effects of others’ behavior partially match the manner in which one’s own predictions, as well as the proprioceptive and affective sensations belonging to self, vary over time. Ambiguity and the associated feelings of a weakening of self–other boundaries, which has been referred to as boundary loss (McNeill, 1997), we-agency (Pacherie, 2011), coupling (Benzon, 2001), and rhythmic entrainment (Becker, 2004), may account for some of the powerful perceptual and emotional responses associated with certain joint behaviors (McLachlan, 2000, 67). Recounting memorable experiences, musicians as well as dancers commonly describe the feeling of oneness with others and the music, of losing themselves and the sense of their body to the moment, and even becoming possessed by an external force (Benzon, 2001, 147). It is notable that, in many cultures, such behaviors are associated with, indeed used to induce, altered states of consciousness, such as trance, in which delusions of control and altered bodily sensations commonly occur (Rouget, 1985; Becker, 1994; Aldridge and Fachner, 2006).

It has been proposed that collective pursuits involving joint action may have evolved to establish and maintain group cohesion (Mithen, 2005; Kirschner and Tomasello, 2010; Reddish et al., 2013; Tarr et al., 2014). This has been supported by findings that associate movement synchrony between individuals with increased feelings of social closeness and affiliation (Decety and Sommerville, 2003; Hove and Risen, 2009; Valdesolo and Desteno, 2011) – a process possibly mediated by the concomitant release of neurohormones such as endorphins (Dunbar et al., 2012; Launay et al., 2016). However, the experience resulting from participating in such joint activities is not typically one of complete and involuntary abandonment. This is because effortful cohesion demands self-awareness and some ability to integrate with others. Indeed, coordinating actions with others to an extent that permits the blurring of boundaries require effective monitoring of self in relation to others to ensure that adjustments of behavior that enable the desired emergent ambiguity to persist. For example, not being able to simultaneously monitor one’s own voice and those of other ensemble members when singing in a choir – completely immersing in the illusion of oneness – is likely to result in going out of tune or out of time with others, reemphasizing the differences that exist between participants. Thus, the performance of joint behaviors requires fluid shifts of attention between monitoring the outcomes of self, other, and the illusory composite sources of self with other.

## Experience-Driven Plasticity of Self

In keeping with findings from research into expert performance (Ericsson et al., 1993), the more time a person spends engaged in activities which challenge accurate self–other attributions, the better that person is likely to become at making such distinctions. If this is the case, individuals with extensive joint-action experience might be expected to be better than average at self–other processing. Music-making experience has already been associated with fundamental behavioral and cognitive changes related to agency that are reflected in functional as well as structural alterations in the brain (reviewed in Jäncke, 2009; Benz et al., 2015). For example, musical training has been associated with more pronounced auditory and motor system coupling (Zatorre et al., 2007), enhanced working memory (e.g., George and Coch, 2011), practice-induced efficiency in motor regions (Jäncke et al., 2000; Chen et al., 2012), and the refinement of cognitive control (e.g., Moreno and Besson, 2006; Helmbold et al., 2005; Lee et al., 2007). Longitudinal and experimental studies suggest that such improvements result from experience and the intensity of training rather than from any innate musical predisposition (e.g., Lahav et al., 2007; Hyde et al., 2009; Moreno et al., 2009; James et al., 2014). In fact, the reliability of such findings has meant that expert musicians have been identified as an ideal cohort to demonstrate mechanisms of experience-driven neuroplasticity (reviewed in Münte et al., 2002).

The idea that music-making develops self-monitoring abilities with observable impacts on brain development may have wider clinical implications. For example, neural changes that result from extensive musical practice could be expected to implicate functions and brain regions that have been highlighted in studies of individuals who report symptoms of schizophrenia, with neural pattern pointing in opposite directions. A direct comparison of musicians and schizophrenia patients could be made with a particular focus on brain regions associated with agency (see, e.g., David et al., 2008). If the ability to accurately differentiate self from other at a perceptual level is malleable and can improve through practice, might such changes impact on the symptoms such as hallucinations and delusions that are specifically thought to be rooted in impairments in self–other processing (Frith et al., 2000; Bentall et al., 2007; Jeannerod, 2009)? To date there is evidence to suggest music’s effectiveness in suppressing symptoms of psychosis (Silverman, 2003; Gold et al., 2009; Na and Yang, 2009; Peng et al., 2010), with musical competence negatively related to symptom severity in schizophrenia (Kantrowitz et al., 2013). There are also precedents for using music as a tool for neuro-rehabilitation (reviewed in Whipple, 2004; Raglio et al., 2008; François et al., 2015).

## Wider Discussion

It is suggested here that a resilient sense of self, essential to mental health, equates to a flexible self and, as such, requires development in contained environments which afford ongoing opportunities for reality-testing. Experience in contexts that sustain a high level of ambiguity and allow individuals to “play” with actual and illusory object boundaries, particularly those that exist between control belonging to self and other, provide optimal conditions for forging a sense of self and provide a buffer to the inevitable individual/internal and environmental changes and stressors that occur throughout life.

Somewhat paradoxically, it may be by weakening self–other and internal–external boundaries – physical and agentic limits and mental attributions – that an individual comes to an enhanced awareness of, and ability to negotiate, perceived shifts in such boundaries. Epstein (1999) suggests that academic psychology has traditionally focused on the individual self as something to be strengthened, without sufficient consideration given to the everyday reality of more fluid, unintegrated states of mind, which have typically only been associated with early infancy (the imaginary friend) and mental illness. As with the young child at play, adult self-awareness routinely vacillates to include states of bodily dissociation, as, for example, when one escapes into the imaginary world of a novel or film (Rochat, 2003). Comparing the Western notion of the self in relation to understandings in Buddhist philosophies, Epstein (1999, 85) contrasts the self as something to be “developed or improved throughout its one-way journey toward separateness” in the former, to the self as variously “expanding and contracting, coalescing and dissolving, separating and merging” in the latter. Milner (2010, 181) concurs that experiences of loss of self, for example as achieved through play, meditation, or absorbed participation in music and arts, should not be dismissed as a regression to an earlier, less developed state, but as a normal phenomenon that enriches perception through the re-encountering of self in novel ways: “there is a plunge into no-differentiation which results (if all goes well) in a re-emerging into a new division of me-not-me, one in which there is more of the ‘me’ in the ‘not-me,’ and more of the ‘not-me’ in the ‘me’.” In this way, playing with realities and non-realities and in the gap between self and non-self, without confusing them lies at the heart of creativity as a dimension of ordinary wellbeing. In contrast to involuntary experiences such as the distressing hallucinations and delusions associated with mental illness, purposive ambiguity-promoting behaviors, involve a deliberative and ultimately contained surrendering of conscious control. The extent to which a participant is able to monitor and influence when and how self disintegrates and/or merges with others may make the difference between an ambiguous context that is playful, creative, and promotes engagement from one that is anxiety-inducing, and potentially annihilating.

Not all pursuits are equal in this regard. With reference to collective activities, Pacherie (2011) provides a helpful distinction between “hierarchical” and “egalitarian” joint action. Using the Western symphony orchestra as an illustration, she argues that in the former, tasks are centralized and specialized, with individual musicians limited in their capacity to control overall outcomes and instead reliant on diktats from the conductor and the constraints imposed by the score/composer. In more egalitarian music systems (often reflecting the collectivist societal structures in the cultures in which these systems tend to emerge), such as African drumming, jazz, and gamelan, governance tends to be distributed across the ensemble. Participants are called upon to predict and monitor individual and combined outcomes, controlling for themselves to differing degrees the variation in coherence and incoherence in relation to one another. This is more reminiscent of the reciprocal dynamic that exists between caregiver–infant interactions where the infant is an active agent and it is safe to momentarily “go to pieces” or merge with the other, “without falling apart” (Epstein, 1999). Thus, distinct forms of music-making or joint action afford qualitatively different subjective experiences of self and other and are likely to mold self-development in diverse ways. In more general terms, although all human interactions involve the need to make behavioral decisions with inadequate information, cultures or communities of practice can promote ambiguity to greater and lesser extents. This may be reflected in the tools and symbols that are used to relate to others. For example, anthropologist Edward Hall (1992) describes languages as being lower or higher context. The former refers to linguistic information that is complete, explicitly contained within the message itself, while a higher context language is one that relies on a greater degree of implicit shared knowledge and interpretation from recipients (Hall, 1992, 229–230). Understanding and questioning the quality of attunement promoted by social, cultural, and political structures, the extent to which practices promote or thward ambiguity between self and other may inform conceptualizations of mental health and strategies put in place for addressing mental illness.

## Conclusion and Future Directions

This paper argues that ambiguity (as fundamental characteristic of many everyday social encounters) plays a key role in developing the sense of self and in learning to differentiate between the boundaries of objects including those that exist between self and other as agents in the world. We propose that engagement in challenging activities that require self–other differentiation may provide optimal conditions for refining reality-testing abilities related to self–other processing. With cross-modal attunement in early infancy framed as one such “healthy” ambiguous context that blurs the boundary between caregiver and infant, the argument positions vulnerability to psychosis-related phenomena within this developmental framework (Bentall et al., 2007). However, the case is also made that attribution competencies negatively associated with hallucinations and delusions in previous research may be malleable and improve through practice. Indeed, certain collective behaviors that put the sense of self into question by introducing a high degree of coherence between autonomous agents (e.g., such as music-making, dance, and certain sports) may be understood as having the functional role of promoting social bonding by improving self–other monitoring capabilities. Experience in ambiguity-promoting contexts may also allow those involved to become better able to tolerate and creatively “play with” modulating self–other sensations, freeing up capacity to refine appropriate reality testing reflexes. This may serve to reduce the likelihood of experiencing unsolicited and distressing misattributions associated with psychosis.

The argument presented here allow specific hypotheses to be generated and tested using behavioral and neuroimaging methods. For example, one over-riding question is whether experience in situations of intricate joint action (such as music-making) positively correlates with the ability to distinguish between action outcomes belonging to self and other, particularly in ambiguous contexts, in contrast to hallucination proneness. What are the neural correlates of this type of ambiguity and of competencies related to self–other differentiation? One might then ask whether improvements in self–other processing, at a perceptual level, generalizes across domains in adulthood. If so, would such improvement impact on phenomena such as hallucinations that have been associated with reduced attribution performance? Related to this are questions around the optimal conditions for bringing about change in self–other processing skills – for example, what types of activities, and constituent elements therein, work best? Such research may not only offer insights into mechanisms mediating the emergence of mental health difficulties, but also has the potential to extend the range of therapeutic possibilities. As Postmes et al. (2014) argue, models of self-disorders such as schizophrenia that focus on lower-level perceptual mechanisms are under-represented in research and clinical literatures. Despite evidence for inter-dependency between top-down and bottom-up processes (e.g., Adcock et al., 2009; Shea, 2014), few therapies target “lower” configurations of self (e.g., core sense of self) that involve more primitive sensations of the body as a coherent entity/agent and its relation to surroundings including other agents over time. We believe that more insight into behavioral and neural responses to ambiguity will contribute to the design of environments and opportunities that maximize such development and will increase our understanding of self-disorders more generally.

## Author Contributions

CdB developed the theoretical formulations and wrote the manuscript with contributions from RS. RC contributed to the final version of the manuscript and supervised the project.

## Conflict of Interest Statement

The authors declare that the research was conducted in the absence of any commercial or financial relationships that could be construed as a potential conflict of interest.

## References

[B1] Abramson L. Y. Seligman M. E. Teasdale J. D. (1978). Learned helplessness in humans: critique and reformulation. *J. Abnorm. Psychol.* 87 49–74. 10.1037/0021-843X.87.1.49 649856

[B2] Adcock R. A. Dale C. Fisher M. Aldebot S. Genevsky A. Simpson G. V. (2009). When top-down meets bottom-up: auditory training enhances verbal memory in schizophrenia. *Schizophr. Bull.* 35 1132–1141. 10.1093/schbul/sbp068 19745022 PMC2762623

[B3] Alais D. Burr D. (2004). The ventriloquist effect results from near-optimal bimodal integration. *Curr. Biol.* 14 257–262. 10.1016/j.cub.2004.01.029 14761661

[B4] Aldridge D. Fachner J. (2006). *Music and Altered States: Consciousness, Transcendence, therapy and Addiction.* London: Jessica Kingsley Publishers.

[B5] Aleman A. (2014). Neurocognitive basis of schizophrenia: information processing abnormalities and clues for treatment. *Adv. Neurosci.* 2014:104920 10.1155/2014/104920

[B6] An S. K. Kang J. I. Park J. Y. Kim K. R. Lee S. Y. Lee E. (2010). Attribution bias in ultra-high risk for psychosis and first-episode schizophrenia. *Schizophr. Res.* 118 54–61. 10.1016/j.schres.2010.01.025 20171849

[B7] Ansfield M. Wegner D. M. (1996). “The feeling of doing,” in *The Psychology of Action: Linking Cognition and Motivation to Behavior* eds Gollwitzer P. M. Bargh J. S. (New York, NY: Guilford) 482–506.

[B8] Badino L. D’Ausilio A. Glowinski D. Camurri A. Fadiga L. (2014). Sensorimotor communication in professional quartets. *Neuropsychologia* 55 98–104. 10.1016/j.neuropsychologia.2013.11.012 24333167

[B9] Bakan M. (2007). *World music: Traditions and Transformations.* New York, NY: McGraw-Hill Higher Education.

[B10] Baron-Cohen S. (1991). “Precursors to a theory of mind: understanding attention in others,” in *Natural theories of Mind: Evolution, Development and Simulation of Everyday Mindreading* ed. Whiten A. (Oxford: Basil Blackwell) 233–251.

[B11] Baumeister R. Bushman B. (2011). “The Self,” in *Social Psychology and Human Nature* 2nd Edn eds Baumeister R. Finkel E. (Belmont, CA: Cengage Learning) 57–96.

[B12] Baumeister R. F. (1987). How the self became a problem: a psychological review of historical research. *J. Pers. Soc. Psychol.* 52 163–176. 10.1037/0022-3514.52.1.163

[B13] Beck A. T. Clark D. A. (1997). An information processing model of anxiety: automatic and strategic processes. *Behav. Res. Ther.* 35 49–58. 10.1016/S0005-7967(96)00069-1 9009043

[B14] Becker J. (1994). Music and trance. *Leonardo Music J.* 4 41–51. 10.2307/1513180

[B15] Becker J. O. (2004). *Deep Listeners: Music, Emotion, and trancing.* Bloomington, IN: Indiana University Press.

[B16] Beebe B. Jaffe J. Markese S. Buck K. Chen H. Cohen P. (2010). The origins of 12-month attachment: a microanalysis of 4-month mother–infant interaction. *Attach. Hum. Dev.* 12 3–141. 10.1080/14616730903338985 20390524 PMC3763737

[B17] Bellack A. S. Morrison R. L. Wixted J. T. Mueser K. T. (1990). An analysis of social competence in schizophrenia. *Br. J. Psychiatry* 156 809–818. 10.1192/bjp.156.6.809 2207511

[B18] Bennett K. Corcoran R. (2010). Biases in everyday reasoning: associations with subclinical anxiety, depression and paranoia. *Psychosis* 2 227–237. 10.1080/17522431003592649

[B19] Benning T. B. (2013). Western and indigenous conceptualizations of self, depression, and its healing. *Int. J. Psychosoc. Rehabil.* 17 129–137.

[B20] Bentall R. P. Fernyhough C. Morrison A. P. Lewis S. Corcoran R. (2007). Prospects for a cognitive-developmental account of psychotic experiences. *Br. J. Clin. Psychol.* 46 155–173. 10.1348/014466506X123011 17524210

[B21] Benz S. Sellaro R. Hommel B. Colzato L. S. (2015). Music makes the world go round: the impact of musical training on non-musical cognitive functions—a review. *Front. Psychol.* 6:2023. 10.3389/fpsyg.2015.02023 26779111 PMC4703819

[B22] Benzon W. (2001). *Beethoven’s Anvil: Music in Mind and Culture.* Oxford: Oxford University Press.

[B23] Berrios G. E. Markova I. S. (2003). “The self and psychiatry: a conceptual history,” in *The Self in Neuroscience and Psychiatry* eds Kircher T. David A. (Cambridge: Cambridge University Press) 9–39. 10.1017/CBO9780511543708.002

[B24] Bion W. R. (1970). *Attention and Interpretation: a Scientific Approach to Insights in Psychoanalysis and Groups.* London: Tavistock.

[B25] Blakemore S. J. Smith J. Steel R. Johnstone E. C. Frith C. D. (2000). The perception of self produced sensory stimuli in patients with auditory hallucinations and passivity experiences: evidence for a break–down in self–monitoring. *Psychol. Med.* 30 1131–1139. 10.1017/S0033291799002676 12027049

[B26] Blakemore S. J. Wolpert D. M. Frith C. D. (2002). Abnormalities in the awareness of action. *Trends Cogn. Sci.* 6 237–242. 10.1016/S1364-6613(02)01907-1 12039604

[B27] Bowlby J. (1973). *Attachment and Loss: Separation* Vol. II. New York, NY: Basic Books.

[B28] Bowlby J. (1982). Attachment and loss: retrospect and prospect. *Am. J. Orthopsychiatry* 52 664–678. 10.1111/j.1939-0025.1982.tb01456.x 7148988

[B29] Braun K. Bock J. (2011). The experience-dependent maturation of prefronto-limbic circuits and the origin of developmental psychopathology: implications for the pathogenesis and therapy of behavioural disorders. *Dev. Med. Child Neurol.* 53 14–18. 10.1111/j.1469-8749.2011.04056.x 21950388

[B30] Brazelton T. B. Koslowski B. Main M. (1974). “The origins ofrec- iprocity: the early mother-infant interaction,” in *The Effect of the Infant on its Caregiver* eds Lewis M. Rosenblum L. A. (New York, NY: Wiley-Interscience) 49–76.

[B31] Bregman A. S. (1994). *Auditory Scene analysis: The Perceptual Organization of Sound.* Cambridge, MA: MIT press.

[B32] Broome M. R. Johns L. C. Valli I. Woolley J. B. Tabraham P. Brett C. (2007). Delusion formation and reasoning biases in those at clinical high risk for psychosis. *Br. J. Psychiatry* 191 s38–s42. 10.1192/bjp.191.51.s38 18055936

[B33] Budner N. Y. (1962). Intolerance of ambiguity as a personality variable1. *J. Pers.* 30 29–50. 10.1111/j.1467-6494.1962.tb02303.x 13874381

[B34] Chen J. L. Rae C. Watkins K. E. (2012). Learning to play a melody: an fMRI study examining the formation of auditory-motor associations. *Neuroimage* 59 1200–1208. 10.1016/j.neuroimage.2011.08.012 21871571

[B35] Christopher J. C. Hickinbottom S. (2008). Positive psychology, ethnocentrism, and the disguised ideology of individualism. *Theory Psychol.* 18 563–589.

[B36] Colbert S. M. Peters E. R. (2002). Need for closure and jumping-to-conclusions in delusion-prone individuals. *J. Nerv. Ment. Dis.* 190 27–31. 10.1177/0959354308093396 11838027

[B37] Combs D. R. Penn D. L. Michael C. O. Basso M. R. Wiedeman R. Siebenmorgan M. (2009). Perceptions of hostility by persons with and without persecutory delusions. *Cogn. Neuropsychiatry* 14 30–52. 10.1080/13546800902732970 19214841

[B38] Corcoran R. Cummins S. Rowse G. Moore R. Blackwood N. Howard R. (2006). Reasoning under uncertainty: heuristic judgments in patients with persecutory delusions or depression. *Psychol. Med.* 36 1109–1118. 10.1080/13546800902732970 16734944

[B39] Corcoran R. Mercer G. Frith C. D. (1995). Schizophrenia, symptomatology and social inference: investigating “theory of mind” in people with schizophrenia. *Schizophr. Res.* 17 5–13. 10.1017/S003329170600794X 8541250

[B40] Critchley H. D. Wiens S. Rotshtein P. Öhman A. Dolan R. J. (2004). Neural systems supporting interoceptive awareness. *Nat. Neurosci.* 7 189–195. 10.1016/0920-9964(95)00024-G 14730305

[B41] Crown C. L. Feldstein S. Jasnow M. D. Beebe B. Jaffe J. (2002). The cross-modal coordination of interpersonal timing: six-week-olds infants’ gaze with adults’ vocal behavior. *J. Psycholinguist. Res.* 31 1–23. 11924837 10.1023/a:1014301303616

[B42] Damasio A. (2012). *Self Comes to Mind: Constructing the Conscious Brain.* New York, NY: Vintage 10.1023/A:1014301303616

[B43] Damasio A. R. (1999). *The Feeling of what Happens: Body and Emotion in the Making of Consciousness.* Boston, MA: Houghton Mifflin Harcourt.

[B44] Damasio A. R. (2003). *Looking for Spinoza: Joy, Sorrow, and the Feeling Brain.* Boston, MA: Houghton Mifflin Harcourt.

[B45] David N. Newen A. Vogeley K. (2008). The “sense of agency” and its underlying cognitive and neural mechanisms. *Conscious. Cogn.* 17 523–534. 10.1016/j.concog.2008.03.004 18424080

[B46] Davis S. (2006). Implied polyphony in the solo string works of JS Bach: a case for the perceptual relevance of structural expression. *Music Percept.* 23 423–446. 10.1525/mp.2006.23.5.423

[B47] Decety J. Sommerville J. A. (2003). Shared representations between self and other: a social cognitive neuroscience view. *Trends Cogn. Sci.* 7 527–533. 10.1016/j.tics.2003.10.004 14643368

[B48] Deneve S. Pouget A. (2004). Bayesian multisensory integration and cross-modal spatial links. *J. Physiol. Paris* 98 249–258. 10.1016/j.jphysparis.2004.03.011 15477036

[B49] Desantis A. Roussel C. Waszak F. (2011). On the influence of causal beliefs on the feeling of agency. *Conscious. Cogn.* 20 1211–1220. 10.1016/j.concog.2011.02.012 21396831

[B50] Ditman T. Kuperberg G. R. (2005). A source-monitoring account of auditory verbal hallucinations in patients with schizophrenia. *Harv. Rev. Psychiatry* 13 280–299. 10.1080/10673220500326391 16251167

[B51] Dowds B. (2014). *Beyond the Frustrated Self: Overcoming Avoidant Patterns and Opening to Life.* London: Karnac Books.

[B52] DunbarR. I. M.KaskatisK.MacDonaldI.BarraV. (2012). Performance of music elevates pain threshold and positive affect: implications for the evolutionary function of music. *Evol. Psychol.* 10 688–702. 10.1177/147470491201000403 23089077

[B53] Epstein H. (1987). *Music Talks: Conversations with Musicians.* New York, NY: McGraw-Hill.

[B54] Epstein M. (1999). *Going to Pieces without Falling Apart: A Buddhist Perspective on Wholeness.* New York, NY: Harmony Books.

[B55] Ericsson K. A. Krampe R. T. Tesch-Römer C. (1993). The role of deliberate practice in the acquisition of expert performance. *Psychol. Rev.* 100 363–406. 10.1037/0033-295X.100.3.363

[B56] Ericsson K. A. Lehmann A. C. (1996). Expert and exceptional performance: evidence of maximal adaptation to task. *Annu. Rev. Psychol.* 47 273–305. 10.1146/annurev.psych.47.1.273 15012483

[B57] Ernst M. O. Bülthoff H. H. (2004). Merging the senses into a robust percept. *Trends Cogn. Sci.* 8 162–169. 10.1016/j.tics.2004.02.002 15050512

[B58] Eysenck M. W. Calvo M. G. (1992). Anxiety and performance: the processing efficiency theory. *Cogn. Emot.* 6 409–434. 10.1080/02699939208409696

[B59] Farrer C. Franck N. (2007). Self-monitoring in schizophrenia. *Curr. Psychiatry Rev.* 3 243–251. 10.2174/157340007782408897

[B60] Farrer C. Franck N. Georgieff N. Frith C. D. Decety J. Jeannerod M. (2003). Modulating the experience of agency: a positron emission tomography study. *Neuroimage* 18 324–333. 10.1016/S1053-8119(02)00041-1 12595186

[B61] Fink D. L. (1988). The core self: a developmental perspective on the dissociative disorders. *Dissociation* 1 43–47. 19167067

[B62] Fonagy P. (2003). The development of psychopathology from infancy to adulthood: the mysterious unfolding of disturbance in time. *Infant Ment. Health J.* 24 212–239. 10.1002/imhj.10053

[B63] Fonagy P. Campbell C. (2015). Bad blood revisited: attachment and psychoanalysis, 2015. *Br. J. Psychother.* 31 229–250. 10.1111/bjp.12150

[B64] Fonagy P. Gergely G. Jurist E. L. (2004). *Affect Regulation, Mentalization and the Development of the Self.* London: Karnac books.

[B65] Fonagy P. Target M. (1996). Playing with reality: I. Theory of mind and the normal development of psychic reality. *Int. J. Psychoanal.* 77 217–234. 8771375

[B66] Forsyth D. R. (1987). *Social Psychology.* Pacific Grove, CA: Brooks/Cole.

[B67] Fosha D. (2001). The dyadic regulation of affect. *J. Clin. Psychol.* 57 227–242. 10.1002/1097-4679(200102)57:2<227::AID-JCLP8>3.0.CO;2-1 11180149

[B68] Franck N. Farrer C. Georgieff N. Marie-Cardine M. Daléry J. d’Amato T. (2001). Defective recognition of one’s own actions in patients with schizophrenia. *Am. J. Psychiatry* 158 454–459. 10.1176/appi.ajp.158.3.454 11229988

[B69] François C. Grau-Sánchez J. Duarte E. Rodriguez-Fornells A. (2015). Musical training as an alternative and effective method for neuro-education and neuro-rehabilitation. *Front. Psychol.* 6:475. 10.3389/fpsyg.2015.00475 25972820 PMC4411999

[B70] Frith C. (2005). The self in action: lessons from delusions of control. *Conscious. Cogn.* 14 752–770. 10.1016/j.concog.2005.04.002 16098765

[B71] Frith C. D. (2007). The social brain? *Philos. Trans. R. Soc. Lond. B Biol. Sci.* 362 671–678. 10.1098/rstb.2006.2003 17255010 PMC1919402

[B72] Frith C. D. Blakemore S. J. Wolpert D. M. (2000). Explaining the symptoms of schizophrenia: abnormalities in the awareness of action. *Brain Res. Rev.* 31 357–363. 10.1016/S0165-0173(99)00052-1 10719163

[B73] Frith C. D. Frith U. (2006). The neural basis of mentalizing. *Neuron* 50 531–534. 10.1016/j.neuron.2006.05.001 16701204

[B74] Gallagher S. (2000). Philosophical conceptions of the self: implications for cognitive science. *Trends Cogn. Sci.* 4 14–21. 10.1016/S1364-6613(99)01417-5 10637618

[B75] Gallagher S. (2005). *How the Body Shapes the Mind.* Oxford: Clarendon Press 173–178. 10.1093/0199271941.001.0001

[B76] Gallagher S. (2013). *Ambiguity in the Sense of Agency.* Oxford: Oxford University Press 118–135. 10.1093/acprof:oso/9780199746996.003.0007

[B77] Gallese V. Sinigaglia C. (2011). What is so special about embodied simulation? *Trends Cogn. Sci.* 15 512–519. 10.1016/j.tics.2011.09.003 21983148

[B78] Gandevia S. C. Burke D. (1992). Does the nervous system depend on kinesthetic information to control natural limb movements? *Behav. Brain Sci.* 15 614–632.

[B79] Garety P. Freeman D. Jolley S. Ross K. Waller H. Dunn G. (2011). Jumping to conclusions: the psychology of delusional reasoning. *Adv. Psychiatr. Treat.* 17 332–339. 10.1192/apt.bp.109.007104

[B80] George E. M. Coch D. (2011). Music training and working memory: an ERP study. *Neuropsychologia* 49 1083–1094. 10.1016/j.neuropsychologia.2011.02.001 21315092

[B81] Gergely G. Fonagy P. Jurist E. Target M. (2002). *Affect Regulation, Mentalization, and the Development of the Self.* New York, NY: Karnac Books.

[B82] Gergely G. Watson J. S. (1996). The social biofeedback model of parental affect-mirroring. *Int. J. Psychoanal.* 77(Pt 6) 1181–1212. 9119582

[B83] Gibson E. J. Pick A. D. (2000). *An Ecological Approach to Perceptual Learning and Development.* New York, NY: Oxford University Press.

[B84] Gibson J. J. (1966). *The Senses Considered as Perceptual Systems.* Boston, MA: Houghton Mifflin.

[B85] Gibson J. J. (1970). On the relation between hallucination and perception. *Leonardo* 3 425–427. 10.2307/1572259

[B86] Gibson J. J. (1979). *The Ecological Approach to Visual Perception.* Boston, MA: Houghton Mifflin.

[B87] Glover N. (2009). *Psychoanalytic Aesthetics: An Introduction to the British School.* London: Karnac Books.

[B88] Gold C. Solli H. P. Krüger V. Lie S. A. (2009). Dose–response relationship in music therapy for people with serious mental disorders: systematic review and meta-analysis. *Clin. Psychol. Rev.* 29 193–207. 10.1016/j.cpr.2009.01.001 19269725

[B89] Green M. F. Horan W. P. (2010). Social cognition in schizophrenia. *Curr. Dir. Psychol. Sci.* 19 243–248. 10.1177/0963721410377600

[B90] Grube M. (2002). Tolerance of ambiguity, art therapy and psychiatric illness. *Psychiatr. Prax.* 29 431–437. 10.1055/s-2002-35509 12436364

[B91] Guignon C. B. (2004). *On Being Authentic.* Hove: Psychology Press.

[B92] Haggard P. (2005). Conscious intention and motor cognition. *Trends Cogn. Sci.* 9 290–295. 10.1016/j.tics.2005.04.012 15925808

[B93] Haggard P. Clark S. Kalogeras J. (2002). Voluntary action and conscious awareness. *Nat. Neurosci.* 5 382–385. 10.1038/nn827 11896397

[B94] Hall E. T. (1992). Improvisation as an acquired, multilevel process. *Ethnomusicology* 36 223–235. 10.2307/851915

[B95] Heider F. (2013). *The Psychology of Interpersonal Relations.* Hove: Psychology Press 10.4324/9780203781159

[B96] Helmbold N. Rammsayer T. Altenmüller E. (2005). Differences in primary mental abilities between musicians and nonmusicians. *J. Individ. Dif.* 26 74–85. 10.1027/1614-0001.26.2.74

[B97] Hove M. J. (2008). Shared circuits, shared time, and interpersonal synchrony. *Behav. Brain Sci.* 31 29–30. 10.1017/S0140525X07003202

[B98] Hove M. J. Risen J. L. (2009). It’s all in the timing: interpersonal synchrony increases affiliation. *Soc. Cogn.* 27 949–960. 10.1521/soco.2009.27.6.949

[B99] Howard I. P. Templeton W. B. (1966). *Human Spatial Orientation.* New York, NY: Wiley.

[B100] Hyde K. L. Lerch J. Norton A. Forgeard M. Winner E. Evans A. C. (2009). Musical training shapes structural brain development. *J. Neurosci.* 29 3019–3025. 10.1523/JNEUROSCI.5118-08.2009 19279238 PMC2996392

[B101] Jaffe J. Beebe B. Feldstein S. Crown C. L. Jasnow M. D. Rochat P. (2001). Rhythms of dialogue in infancy: coordinated timing in development. *Monogr. Soc. Res. Child Dev.* 66 i–viii, 1–132. 11428150

[B102] James C. E. Oechslin M. S. Van De Ville D. Hauert C. A. Descloux C. Lazeyras F. (2014). Musical training intensity yields opposite effects on grey matter density in cognitive versus sensorimotor networks. *Brain Struct. Funct.* 219 353–366. 10.1007/s00429-013-0504-z 23408267

[B103] James W. (1891). *The Principles of Psychology* Vol. 1 Cambridge, MA: Harvard University Press

[B104] Jäncke L. (2009). The plastic human brain. *Restor. Neurol. Neurosci.* 27 521–538. 10.3233/RNN-2009-0519 19847074

[B105] Jäncke L. Shah N. J. Peters M. (2000). Cortical activations in primary and secondary motor areas for complex bimanual movements in professional pianists. *Cogn. Brain Res.* 10 177–183. 10.1016/S0926-6410(00)00028-8 10978706

[B106] Jeannerod M. (2003). The mechanism of self-recognition in humans. *Behav. Brain Res.* 142 1–15. 10.1016/S0166-4328(02)00384-4 12798261

[B107] Jeannerod M. (2009). The sense of agency and its disturbances in schizophrenia: a reappraisal. *Exp. Brain Res.* 192 527–532. 10.1007/s00221-008-1533-3 18709365

[B108] Jeon I. H. Kim K. R. Kim H. H. Park J. Y. Lee M. Jo H. H. (2013). Attributional style in healthy persons: its association with ‘theory of mind’ skills. *Psychiatry Investig.* 10 34–40. 10.4306/pi.2013.10.1.34 23482524 PMC3590428

[B109] Jonsson C. O. Clinton D. Fahrman M. Mazzaglia G. Novak S. Sörhus K. (2001). How do mothers signal shared feeling-states to their infants? An investigation of affect attunement and imitation during the first year of life. *Scand. J. Psychol.* 42 377–381. 10.1111/1467-9450.00249 11547913

[B110] Kantrowitz J. T. Leitman D. I. Lehrfeld J. M. Laukka P. Juslin P. N. Butler P. D. (2013). Reduction in tonal discriminations predicts receptive emotion processing deficits in schizophrenia and schizoaffective disorder. *Schizophr. Bull.* 39 86–93. 10.1093/schbul/sbr060 21725063 PMC3523919

[B111] Kapur S. (2003). Psychosis as a state of aberrant salience: a framework linking biology, phenomenology, and pharmacology in schizophrenia. *Am. J. Psychiatry* 160 13–23. 10.1176/appi.ajp.160.1.13 12505794

[B112] Kassin S. Fein S. Markus H. (2010). *Social Psychology* 8th Edn. Wadsworth, OH: Cengage Learning.

[B113] Keller T. A. Just M. A. (2016). Structural and functional neuroplasticity in human learning of spatial routes. *Neuroimage* 125 256–266. 10.1016/j.neuroimage.2015.10.015 26477660

[B114] Kelley H. H. (1967). *Attribution theory in Social Psychology. In Nebraska Symposium on Motivation.* Lincoln, NE: University of Nebraska Press.

[B115] Kircher T. David A. (2003). *The Self in Neuroscience and Psychiatry.* Cambridge: Cambridge University Press 10.1017/CBO9780511543708

[B116] Kirschner S. Tomasello M. (2010). Joint music making promotes prosocial behavior in 4-year-old children. *Evol. Hum. Behav.* 31 354–364. 10.1016/j.evolhumbehav.2010.04.004

[B117] Klein S. B. (2012). Self, memory, and the self-reference effect an examination of conceptual and methodological issues. *Pers. Soc. Psychol. Rev.* 16 283–300. 10.1177/1088868311434214 22291045

[B118] Klein S. B. Gangi C. E. (2010). The multiplicity of self: neuropsychological evidence and its implications for the self as a construct in psychological research. *Ann. N. Y. Acad. Sci.* 1191 1–15. 10.1111/j.1749-6632.2010.05441.x 20392272

[B119] Kolb B. Mychasiuk R. Muhammad A. Li Y. Frost D. O. Gibb R. (2012). Experience and the developing prefrontal cortex. *Proc. Natl. Acad. Sci. U.S.A.* 109(Suppl. 2) 17186–17193. 10.1073/pnas.1121251109 23045653 PMC3477383

[B120] Konvalinka I. Vuust P. Roepstorff A. Frith C. D. (2010). Follow you, follow me: continuous mutual prediction and adaptation in joint tapping. *Q. J. Exp. Psychol.* 63 2220–2230. 10.1080/17470218.2010.497843 20694920

[B121] Kranick S. M. Hallett M. (2013). Neurology of volition. *Exp. Brain Res.* 229 313–327. 10.1007/s00221-013-3399-2 23329204 PMC4744643

[B122] Kuhl P. K. Meltzoff A. N. (1982). The bimodal perception of speech in infancy. *Science* 218 1138–1141. 10.1126/science.7146899 7146899

[B123] Kyrios M. Nelson B. Ahern C. Fuchs T. Parnas J. (2015). The self in psychopathology. *Psychopathology* 48 275–277. 10.1159/000438876 26372951

[B124] Lahav A. Saltzman E. Schlaug G. (2007). Action representation of sound: audiomotor recognition network while listening to newly acquired actions. *J. Neurosci.* 27 308–314. 10.1523/JNEUROSCI.4822-06.2007 17215391 PMC6672064

[B125] Launay J. Tarr B. Dunbar R. I. (2016). Synchrony as an adaptive mechanism for large-scale human social bonding. *Ethology* 122 779–789. 10.1111/eth.12528

[B126] Leary M. R. Tangney J. P. (eds) (2011). *Handbook of Self and Identity.* New York, NY: Guilford Press.

[B127] Lee Y. S. Lu M. J. Ko H. P. (2007). Effects of skill training on working memory capacity. *Learn. Instruct.* 17 336–344. 10.1016/j.learninstruc.2007.02.010

[B128] Lewkowicz D. J. Turkewitz G. (1980). Cross-modal equivalence in early infancy: auditory–visual intensity matching. *Dev. Psychol.* 16 597–607. 10.1037/0012-1649.16.6.597

[B129] Lieberman M. D. (2007). Social cognitive neuroscience: a review of core processes. *Annu. Rev. Psychol.* 58 259–289. 10.1146/annurev.psych.58.110405.085654 17002553

[B130] Lindestad P. Å. Södersten M. Merker B. Granqvist S. (2001). Voice source characteristics in Mongolian “throat singing” studied with high-speed imaging technique, acoustic spectra, and inverse filtering. *J. Voice* 15 78–85. 10.1016/S0892-1997(01)00008-X 12269637

[B131] Lindner A. Thier P. Kircher T. T. Haarmeier T. Leube D. T. (2005). Disorders of agency in schizophrenia correlate with an inability to compensate for the sensory consequences of actions. *Curr. Biol.* 15 1119–1124. 10.1016/j.cub.2005.05.049 15964277

[B132] Linney Y. M. Peters E. R. Ayton P. (1998). Reasoning biases in delusion–prone individuals. *Br. J. Clin. Psychol.* 37 285–302. 10.1111/j.2044-8260.1998.tb01386.x 9784884

[B133] Malloch S. Trevarthen C. (2009). “Musicality: communicating the vitality and interests of life,” in *Communicative Musicality: Exploring the Basis of Human Companionship* Vol. 1 eds Malloch S. Trevarthen C. (Oxford: Oxford University Press) 1–10.

[B134] Maule A. J. Hockey G. R. J. Bdzola L. (2000). Effects of time-pressure on decision-making under uncertainty: changes in affective state and information processing strategy. *Acta Psychol.* 104 283–301. 10.1016/S0001-6918(00)00033-0 10900697

[B135] McLachlan N. (2000). A spatial theory of rhythmic resolution. *Leonardo Music J.* 10 61–67. 10.1162/096112100570468

[B136] McNeill W. H. (1997). *Keeping Together in Time.* Cambridge, MA: Harvard University Press.

[B137] Meltzoff A. N. Moore M. K. (1995). “Infants’ understanding of people and things: from body imitation to folk psychology,” in *The Body and the Self* eds Bermúdez J. L. Marcel A. J. Eilan N. (Cambridge, MA: The MIT Press) 43–69.

[B138] Milner M. (2010). *On not Being able to Paint.* Abingdon: Routledge 10.4324/9780203833650

[B139] Mitchell J. P. Banaji M. R. MacRae C. N. (2005). The link between social cognition and self-referential thought in the medial prefrontal cortex. *J. Cogn. Neurosci.* 17 1306–1315. 10.1162/0898929055002418 16197685

[B140] Mithen S. (2005). *The Singing Neanderthals: The Origin of Language, Music, Mind and Body.* London: Weidenfeld and Nicolson.

[B141] Moore J. W. Fletcher P. C. (2012). Sense of agency in health and disease: a review of cue integration approaches. *Conscious. Cogn.* 21 59–68. 10.1016/j.concog.2011.08.010 21920777 PMC3315009

[B142] Moore J. W. Wegner D. M. Haggard P. (2009). Modulating the sense of agency with external cues. *Conscious. Cogn.* 18 1056–1064. 10.1016/j.concog.2009.05.004 19515577

[B143] Moreno S. Besson M. (2006). Musical training and language-related brain electrical activity in children. *Psychophysiology* 43 287–291. 10.1111/j.1469-8986.2006.00401.x 16805867

[B144] Moreno S. Marques C. Santos A. Santos M. Castro S. L. Besson M. (2009). Musical training influences linguistic abilities in 8-year-old children: more evidence for brain plasticity. *Cereb. Cortex* 19 712–723. 10.1093/cercor/bhn120 18832336

[B145] Moskowitz G. B. (2005). *Social Cognition: Understanding Self and others.* New York, NY: Guilford Press.

[B146] Münte T. F. Altenmüller E. Jäncke L. (2002). The musician’s brain as a model of neuroplasticity. *Nat. Rev. Neurosci.* 3 473–478. 10.1038/nrn843 12042882

[B147] Myers D. G. Smith S. M. (2012). *Exploring Social Psychology.* New York, NY: McGraw-Hill.

[B148] Na H. J. Yang S. (2009). Effects of listening to music on auditory hallucination and psychiatric symptoms in people with schizophrenia. *J. Korean Acad. Nurs.* 39 62–71. 10.4040/jkan.2009.39.1.62 19265313

[B149] Nahab F. B. Kundu P. Gallea C. Kakareka J. Pursley R. Pohida T. (2011). The neural processes underlying self-agency. *Cereb. Cortex* 21 48–55. 10.1093/cercor/bhq059 20378581 PMC3000563

[B150] Overy K. Molnar-Szakacs I. (2009). Being together in time: musical experience and the mirror neuron system. *Music Percept.* 26 489–504. 10.1371/journal.pone.0013812 21179549 PMC3002933

[B151] Pacherie E. (2011). “The phenomenology of joint action: self-agency versus joint agency,” in *Joint Attention: New Developments in Psychology, Philosophy of Mind, and Social Neuroscience* ed. Seemann A. (Cambridge, MA: MIT Press) 343–389.

[B152] Palmer S. E. (2003). “Visual perception of objects,” in *Handbook of Psychology: Experimental Psychology 4* eds Healy A. F. Proctor R. W. Weiner I. B. (Hoboken, NJ: John Wiley and Sons).

[B153] Parnas J. Handest P. (2003). Phenomenology of anomalous self-experience in early schizophrenia. *Compr. Psychiatry* 44 121–134. 10.1053/comp.2003.50017 12658621

[B154] Pascual-Leone A. Freitas C. Oberman L. Horvath J. C. Halko M. Eldaief M. (2011). Characterizing brain cortical plasticity and network dynamics across the age-span in health and disease with TMS-EEG and TMS-fMRI. *Brain Topogr.* 24 302–315. 10.1007/s10548-011-0196-8 21842407 PMC3374641

[B155] Pascual-Leone A. Hamilton R. (2001). The metamodal organization of the brain. *Prog. Brain Res.* 134 427–446. 10.1016/S0079-6123(01)34028-1 11702559

[B156] Peng S. M. Koo M. Kuo J. C. (2010). Effect of group music activity as an adjunctive therapy on psychotic symptoms in patients with acute schizophrenia. *Arch. Psychiatr. Nurs.* 24 429–434. 10.1016/j.apnu.2010.04.001 21111297

[B157] Perry B. D. (2002). Childhood experience and the expression of genetic potential: what childhood neglect tells us about nature and nurture. *Brain Mind* 3 79–100. 10.1023/A:1016557824657

[B158] Ponti G. Peretto P. Bonfanti L. (2008). Genesis of neuronal and glial progenitors in the cerebellar cortex of peripuberal and adult rabbits. *PLoS One* 3:e2366. 10.1371/journal.pone.0002366 18523645 PMC2396292

[B159] Postmes L. Sno H. N. Goedhart S. van der Stel J. Heering H. D. de Haan L. (2014). Schizophrenia as a self-disorder due to perceptual incoherence. *Schizophr. Res.* 152 41–50. 10.1016/j.schres.2013.07.027 23973319

[B160] Poulet J. F. Hedwig B. (2007). New insights into corollary discharges mediated by identified neural pathways. *Trends Neurosci.* 30 14–21. 10.1016/j.tins.2006.11.005 17137642

[B161] Premack D. Woodruff G. (1978). Does the chimpanzee have a theory of mind? *Behav. Brain Sci.* 1 515–526. 10.1017/S0140525X00076512

[B162] Pyszczynski T. Greenberg J. (1987). Self-regulatory perseveration and the depressive self-focusing style: a self-awareness theory of reactive depression. *Psychol. Bull.* 102 122–138. 10.1037/0033-2909.102.1.122 3615702

[B163] Raglio A. Bellelli G. Traficante D. Gianotti M. Ubezio M. C. Villani D. (2008). Efficacy of music therapy in the treatment of behavioral and psychiatric symptoms of dementia. *Alzheimer Dis. Assoc. Disord.* 22 158–162. 10.1097/WAD.0b013e3181630b6f 18525288

[B164] Ramachandran V. S. Hirstein W. (1999). The science of art: a neurological theory of aesthetic experience. *J. Conscious. Stud.* 6 15–51.

[B165] Ramachandran V. S. Rogers-Ramachandran D. (2008). Ambiguities & perception. *Sci. Am.* 18 56–59. 10.1038/scientificamerican0508-56sp

[B166] Reddish P. Fischer R. Bulbulia J. (2013). Let’s dance together: synchrony, shared intentionality and cooperation. *PLoS One* 8:e71182. 10.1371/journal.pone.0071182 23951106 PMC3737148

[B167] Rimmon-Kenan S. (1977). *The Concept of Ambiguity–the Example of James.* Chicago, IL: University of Chicago Press.

[B168] Roach N. W. Heron J. McGraw P. V. (2006). Resolving multisensory conflict: a strategy for balancing the costs and benefits of audio-visual integration. *Proc. R. Soc. Lond. B Biol. Sci.* 273 2159–2168. 10.1098/rspb.2006.3578 16901835 PMC1635528

[B169] Rochat P. (1998). Self-perception and action in infancy. *Exp. Brain Res.* 123 102–109. 10.1007/s002210050550 9835398

[B170] Rochat P. (2003). Five levels of self-awareness as they unfold early in life. *Conscious. Cogn.* 12 717–731. 10.1016/S1053-8100(03)00081-3 14656513

[B171] Rock I. Palmer S. (1990). Gestalt psychology. *Sci. Am.* 263 84–90. 10.1038/scientificamerican1290-84 2270461

[B172] Rogers C. (1961). *On Becoming a Person: A Therapist’s view of Psychotherapy.* London: Constable.

[B173] Rose G. J. (2004). *Between Couch and Piano: Psychoanalysis, Music, Art and Neuroscience.* Abingdon: Routledge 10.4324/9780203462904

[B174] Rouget G. (1985). *Music and Trance: A theory of the Relations Between Music and Possession.* Chicago, IL: University of Chicago Press.

[B175] Sass L. A. Parnas J. (2003). Schizophrenia, consciousness, and the self. *Schizophr. Bull.* 29 427–444. 10.1093/oxfordjournals.schbul.a007017 14609238

[B176] Sato A. Yasuda A. (2005). Illusion of sense of self–agency: discrepancy between the predicted and actual sensory consequences of actions modulates the sense of self–agency, but not the sense of self–ownership. *Cognition* 94 241–255. 10.1016/j.cognition.2004.04.003 15617673

[B177] Savla G. N. Vella L. Armstrong C. C. Penn D. L. Twamley E. W. (2012). Deficits in domains of social cognition in schizophrenia: a meta-analysis of the empirical evidence. *Schizophr. Bull.* 39 979–992. 10.1093/schbul/sbs080 22949733 PMC3756768

[B178] Schore A. N. (2003). *Affect Dysregulation and Disorders of the Self.* New York, NY: Norton.

[B179] Schore A. N. (2015). *Affect Regulation and the Origin of the Self: The Neurobiology of Emotional Development.* Abingdon: Routledge 10.4324/9781315680019

[B180] Sebanz N. Bekkering H. Knoblich G. (2006). Joint action: bodies and minds moving together. *Trends Cogn. Sci.* 10 70–76. 10.1016/j.tics.2005.12.009 16406326

[B181] Shea N. (2014). “Distinguishing top-down from bottom-up effects,” in *Perception and its Modalities* eds Biggs S. Matthen M. Stokes D. (Oxford: Oxford University Press) 73–91.

[B182] Siegel D. J. (2001). Toward an interpersonal neurobiology of the developing mind: attachment relationships, “mindsight,” and neural integration. *Infant Ment. Health J.* 22 67–94. 10.1002/1097-0355(200101/04)22:1<67::AID-IMHJ3>3.0.CO;2-G

[B183] Silverman M. J. (2003). The influence of music on the symptoms of psychosis: a meta-analysis. *J. Music Ther.* 40 27–40. 10.1093/jmt/40.1.27 17590966

[B184] Sloboda A. (2000). Individual differences in music performance. *Trends Cogn. Sci.* 4 397–403. 10.1016/S1364-6613(00)01531-X 11025283

[B185] Soto-Faraco S. Lyons J. Gazzaniga M. Spence C. Kingstone A. (2002). The ventriloquist in motion: illusory capture of dynamic information across sensory modalities. *Cogn. Brain Res.* 14 139–146. 10.1016/S0926-6410(02)00068-X 12063137

[B186] Spence S. A. Brooks D. J. Hirsch S. R. Liddle P. F. Meehan J. Grasby P. M. (1997). A PET study of voluntary movement in schizophrenic patients experiencing passivity phenomena (delusions of alien control). *Brain* 120 1997–2011. 10.1093/brain/120.11.1997 9397017

[B187] Sperduti M. Delaveau P. Fossati P. Nadel J. (2011). Different brain structures related to self-and external-agency attribution: a brief review and meta-analysis. *Brain Struct. Funct.* 216 151–157. 10.1007/s00429-010-0298-1 21212978

[B188] Stein B. E. Meredith M. A. (1993). *The Merging of the Senses.* Cambridge, MA: The MIT Press.

[B189] Stern D. N. (1985). *The Interpersonal World of the Infant. A View from Psychoanalysis and Developmental Psychology.* New York, NY: Basic Books.

[B190] Stern D. N. (2010). *Forms of Vitality: Exploring Dynamic Experience in Psychology, the Arts, Psychotherapy, and Development.* Oxford: Oxford University Press 10.1093/med:psych/9780199586066.001.0001

[B191] Stuss D. T. Gallup G. G. Alexander M. P. (2001). The frontal lobes are necessary for theory of mind’. *Brain* 124 279–286. 10.1093/brain/124.2.279 11157555

[B192] Sutton-Smith B. (2009). *The Ambiguity of Play.* Cambridge, MA: Harvard University Press.

[B193] Synofzik M. Vosgerau G. Newen A. (2008). Beyond the comparator model: a multifactorial two-step account of agency. *Conscious. Cogn.* 17 219–239. 10.1016/j.concog.2007.03.010 17482480

[B194] Tarr B. Launay J. Dunbar R. I. (2014). Music and social bonding: “self-other” merging and neurohormonal mechanisms. *Front. Psychol.* 5:1096. 10.3389/fpsyg.2014.01096 25324805 PMC4179700

[B195] Tekin S. Cummings J. L. (2002). Frontal–subcortical neuronal circuits and clinical neuropsychiatry: an update. *J. Psychosom. Res.* 53 647–654. 10.1016/S0022-3999(02)00428-2 12169339

[B196] Tenzer M. (1998). *Balinese Music.* Clarendon, VT: Tuttle Publishing.

[B197] Trinity B. Sommer M. (2008). Corollary discharge across the animal kingdom. *Nat. Rev. Neurosci.* 9 587–600. 10.1038/nrn2457 18641666 PMC5153363

[B198] Tronick E. (2007). *The Neurobehavioral and Social-Emotional Development of Infants and Children.* New York, NY: WW Norton & Company.

[B199] Tsai C. G. (2004). The timbre space of the Chinese membrane flute (Dizi): physical basis and psychoacoustical effects. *J. Acoust. Soc. Am.* 116 2620–2620. 10.1121/1.4785447

[B200] Uddin L. Q. Iacoboni M. Lange C. Keenan J. P. (2007). The self and social cognition: the role of cortical midline structures and mirror neurons. *Trends Cogn. Sci.* 11 153–157. 10.1016/j.tics.2007.01.001 17300981

[B201] Valdesolo P. Desteno D. (2011). Synchrony and the social tuning of compassion. *Emotion* 11 262–266. 10.1037/a0021302 21500895

[B202] Van Dael F Versmissen D. Janssen I. Myin-Germeys I. van Os J Krabbendam L. (2006). Data gathering: biased in psychosis? *Schizophr. Bull.* 32 341–351. 16254066 10.1093/schbul/sbj021 PMC2632225

[B203] Von Holst E. (1954). Relations between the central nervous system and the peripheral organs. *Br. J. Anim. Behav.* 2 89–94. 10.1016/S0950-5601(54)80044-X

[B204] Wegner D. M. (2002). *The Illusion of Conscious Will.* Cambridge, MA: MIT Press.

[B205] Wegner D. M. (2003). The mind’s best trick: how we experience conscious will. *Trends Cogn. Sci.* 7 65–69. 10.1016/S1364-6613(03)00002-0 12584024

[B206] Wegner D. M. Sparrow B. Winerman L. (2004). Vicarious agency: experiencing control over the movements of others. *J. Pers. Soc. Psychol.* 86 838–848. 10.1037/0022-3514.86.6.838 15149258

[B207] Wernery J. (2013). *Bistable Perception of the Necker Cube in the Context of Cognition & Personality.* Ph.D. dissertation, Zürich, University of Zürich.

[B208] Whipple J. (2004). Music in intervention for children and adolescents with autism: a meta-analysis. *J. Music Ther.* 41 90–106. 10.1093/jmt/41.2.90 15307805

[B209] White P. A. (1995). *The Understanding of Causation and the Production of Action: From Infancy to Adulthood.* Hove: Psychology Press.

[B210] Winnicott D. W. (1960). The theory of the parent-infant relationship. *Int. J. Psychoanal.* 41 585–595. 13785877

[B211] Winnicott D. W. (1971). *Playing and Reality.* Hove: Psychology Press.

[B212] Winton-Brown T. T. Fusar-Poli P. Ungless M. A. Howes O. D. (2014). Dopaminergic basis of salience dysregulation in psychosis. *Trends Neurosci.* 37 85–94. 10.1016/j.tins.2013.11.003 24388426

[B213] Wolpert D. M. Miall R. C. (1996). Forward models for physiological motor control. *Neural Netw.* 9 1265–1279. 10.1016/S0893-6080(96)00035-4 12662535

[B214] Wood J. N. Grafman J. (2003). Human prefrontal cortex: processing and representational perspectives. *Nat. Rev. Neurosci.* 4 139–147. 10.1038/nrn1033 12563285

[B215] Woodruff P. W. (2004). Auditory hallucinations: insights and questions from neuroimaging. *Cogn. Neuropsychiatry* 9 73–91. 10.1080/13546800344000165 16571575

[B216] Zatorre R. J. Chen J. L. Penhune V. B. (2007). When the brain plays music: auditory-motor interactions in music perception and production. *Nat. Rev. Neurosci.* 8 547–558. 17585307 10.1038/nrn2152

